# An Indoor Positioning Method for Smartphones Using Landmarks and PDR †

**DOI:** 10.3390/s16122135

**Published:** 2016-12-15

**Authors:** Xi Wang, Mingxing Jiang, Zhongwen Guo, Naijun Hu, Zhongwei Sun, Jing Liu

**Affiliations:** 1 Department of Computer Science and Technology, Ocean University of China, Qingdao 266100, China; abwangxi@163.com (X.W.); sunzhongwei0423@126.com (Z.S.); liuj608@163.com (J.L.); 2 Department of Computer Foundation, Ocean University of China, Qingdao 266100, China; jiangmx@ouc.edu.cn; 3 Information Center, Administration for Industry and Commerce of Qingdao, Qingdao 266071, China; hunaijun@sina.com

**Keywords:** indoor positioning, PDR, landmarks, fusion

## Abstract

Recently location based services (LBS) have become increasingly popular in indoor environments. Among these indoor positioning techniques providing LBS, a fusion approach combining WiFi-based and pedestrian dead reckoning (PDR) techniques is drawing more and more attention of researchers. Although this fusion method performs well in some cases, it still has some limitations, such as heavy computation and inconvenience for real-time use. In this work, we study map information of a given indoor environment, analyze variations of WiFi received signal strength (RSS), define several kinds of indoor landmarks, and then utilize these landmarks to correct accumulated errors derived from PDR. This fusion scheme, called Landmark-aided PDR (LaP), is proved to be light-weight and suitable for real-time implementation by running an Android application designed for the experiment. We compared LaP with other PDR-based fusion approaches. Experimental results show that the proposed scheme can achieve a significant improvement with an average accuracy of 2.17 m.

## 1. Introduction

Recently, location based services (LBS) are becoming increasingly popular in indoor environments because massive wireless networks are built according to the IEEE 802.11 wireless Ethernet standard. Indoor mobile positioning techniques are the backbone of LBS. These techniques can be generally divided into two categories according to different measurements adopted: pedestrian dead reckoning (PDR) based on inertial sensors such as accelerometers, gyroscopes, etc. [1]; and location determination employing received signal strength (RSS) of WiFi as a metric [2].

PDR is a self-contained approach but will produce a growing drift as walking distance increases [3]. It relies on readings of inertial sensors embedded in smartphones to detect steps, calculate step length and determine walking direction. RSS-based positioning mainly includes the model-based approach and fingerprinting method. Because of background interference, non-uniform spreading, signal fading and reflections in WiFi signal propagation [4], accurate path-loss model is hard to obtain, thus leading to an inevitable distinct error. The fingerprinting method has a higher accuracy, but requires tedious manual collection of data for training before positioning. In short, these RSS-based approaches are not suitable to implement on smartphones, either because of their low accuracy, or because of complicated preprocessing. Recent research tends to combine both PDR and RSS-based techniques to achieve a better performance [5,6,7]. Indoor map information is also taken into consideration among some of these studies [8,9].

Through observations, we find human behaviors, like turning, going upstairs or downstairs, can be easily recognized by reading value variations of inertial sensors on smartphones, such as gyroscopes, altimeter sensors, and accelerometers, etc. In addition, we also notice that obvious fluctuation of WiFi RSS will take place when pedestrians pass by doors, or across projection points of WiFi Access Points (AP) in their walking paths. Such locations of turns, doors, and AP projection points can be obtained accurately when map information is available, and they can be used to determine locations of the pedestrians. These locations are supposed to be identified in real time while pedestrians are walking, and will then be regarded as landmarks in order to be new initial points of a PDR algorithm to eliminate system cumulative errors.

In this paper, we study the map information, analyze variations of inertial sensor value on smartphones, determine locations of landmarks in a real-time way, and then propose an efficient, feasible fusion scheme for combining landmarks and PDR on smartphones, called Landmark-aided PDR (LaP). Based on this fusion scheme, an indoor positioning system is established to provide positions of pedestrians in a real-time way without previous WiFi fingerprinting store. A comparison has been made among LaP and other PDR-based fusion approaches. Experimental results show that the proposed scheme can improve overall performance significantly with an average accuracy of 2.17 m.

The rest of the paper is organized as follows: some related works in the literature are discussed in Section 2. In Section 3, we present a conventional PDR approach and methods of identifying landmarks, as well as the fusion scheme. Section 4 is an evaluation and discussion of the experimental results. We present conclusions of the paper and reveal some potential future works in Section 5.

## 2. Related Work

A conventional PDR mainly contains three parts: step detection, step length estimation and walking direction estimation. For step detection, there is a common method called peak detection [10,11], which can be used to analyze acceleration signals. Authors gave a dynamic step length estimation method based on proportional relationship between hip bounce and step length [12]. An experimental equation representing a relation between step length and average acceleration during a step was proposed by Jeong Won Kim et al. [10]. For the walking direction estimation, some of the recent research focuses on combining gyroscopes with other inertial sensors such as geomagnetic sensors [13,14,15].

There are accumulated errors in PDR due to the drift of inertial sensors. Integrating PDR with other positioning methods is a better solution to achieve high positioning accuracy. Recent research tends to fuse RSS-based localization technique and PDR together to navigate the pedestrian in indoor environments [5,6,7]. Most of the existing fusion methods adopt the Kalman filter or particle filter. In addition, they almost require a pre-collection of RSS, which makes positioning redundant and impractical. Both Guo Chen et al. [5] and Yu Li et al. [6] utilized the relationship between distance and RSS to achieve a better positioning result based on PDR. However, a full understanding of the indoor environment is needed to formulate an accurate mapping relation of distance and RSS, which usually is an arduous task. In the work of W. Xiao et al. [7], a stochastic system model is adopted to track the target’s position via an inertial measurement unit integrated with the WiFi tag. To overcome the severe signal instability problem in the indoor environment, in the work of Jenq-Shiou Leu et al. [16], an RSS fingerprint and footprint matching mechanism with the assistance of collecting ambient WiFi RSSs from not only the intrinsic APs but also the extrinsic APs is proposed. All of the WiFi fingerprint-based approaches achieved a better positioning performance, but the fingerprint acquisition work is needed in advance.

Floor plans are also valuable references in indoor mobile positioning and can be combined with PDR. K.C. Lan et al. transformed the floor plan into a link model, based on which a trajectory-based map matching algorithm was proposed [17]. K.W. Chiang et al. put the focus on a fuzzy decision tree aided by map information to improve the accuracy and stability of PDR [18]. I. Miller et al. proposed an indoor positioning scheme by fusing floor map and smartphone sensor data without additional infrastructure [19]. For this literature focusing on combinations of floor plans and PDR, a better performance is probably achieved if a WiFi based approach is adopted at the same time.

Since only WiFi + PDR or floor plan + PDR-based indoor fusion positioning cannot provide a satisfying result, some researchers focus on the fusion of WiFi, PDR and floor plans at the same time. Chen et al. proposed a sensor fusion framework for combining WiFi, PDR and floor plans [20], and the fusion was implemented by using a Kalman filter. To deal with WiFi-based localization, they applied a weighted pass loss (WPL) algorithm. Meanwhile, landmarks are introduced to tackle the initial estimation error of the PDR-based approach.

Similar fusion algorithm can be found in the work of Wang et al. [21]. They proposed a scheme for indoor positioning by fusing a floor map, WiFi and smartphone sensor data. A topology-constrained k-nearest neighbor (KNN) algorithm based on a floor map layout provided the coordinates required to integrate WiFi data with pseudo-odometry (P-O) measurements. The combination of all three techniques was implemented by using a particle filter (PF) model.

One of the most recent works can be found in the work of Deng et al. [22], in which, similar to other works, the authors also utilized WiFi, PDR and a floor plan at the same time. The innovation was that they used an extended Kalman filter (EKF) twice, and during the WiFi positioning phase, they adopted a kernel density estimation (KDE) model. Experimental results in a realistic indoor environment showed that the proposed fusion approach achieved substantial positioning accuracy improvement compared with individual positioning approaches including PDR and WiFi positioning.

## 3. Proposed LaP System

### 3.1. System Overview

**Figure 1:**
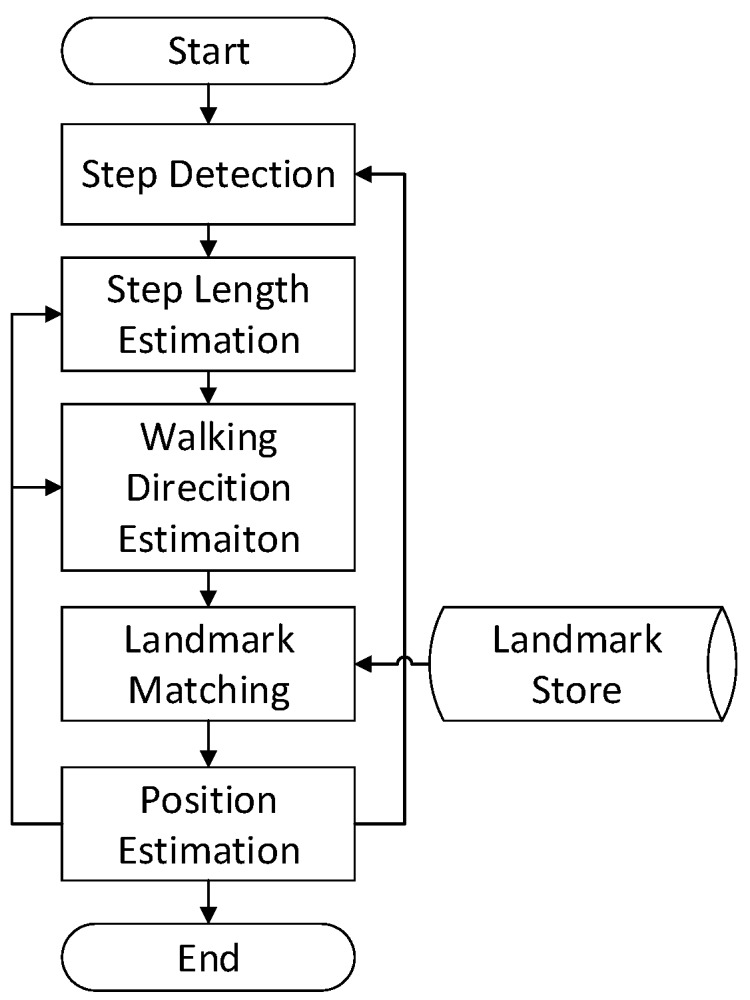
Flow chart of LaP.

All localization techniques have their own strengths and drawbacks, including the PDR approach: providing high accuracy within a short range but leading to an inevitable drift during a pedestrian walk. In this section, we will give an introduction of a conventional PDR algorithm, redefine the term ’landmarks’, and then correct the drift error with these landmarks. The flow chart of the proposed LaP is shown in Figure 1.

### 3.2. PDR Subsystem

PDR is a pedestrian positioning solution that determines the next position of a pedestrian by adding travelled distance to the previous position, as Equation (1) shows:

(1) $$P_{t}=P_{t-1}+D_{t}\left(\begin{array}{c} \sin(\varphi_{t})\\ \cos(\varphi_{t}) \end{array}\right),$$

where $P_{t}$ is the position at time stamp *t*, $D_{t}$ is the step length and $\varphi_{t}$ is the walking direction at time stamp *t*.

Current off-the-shelf inertial sensors, such as accelerometers, magnetometers and gyroscopes, become more trustworthy and are widely embedded in smartphones, so a PDR system can be implemented in these intelligent terminals more reliably. A classic PDR mainly contains three parts: step detection, step length estimation and walking direction estimation.

#### 3.2.1. Step Detection

**Figure 2:**
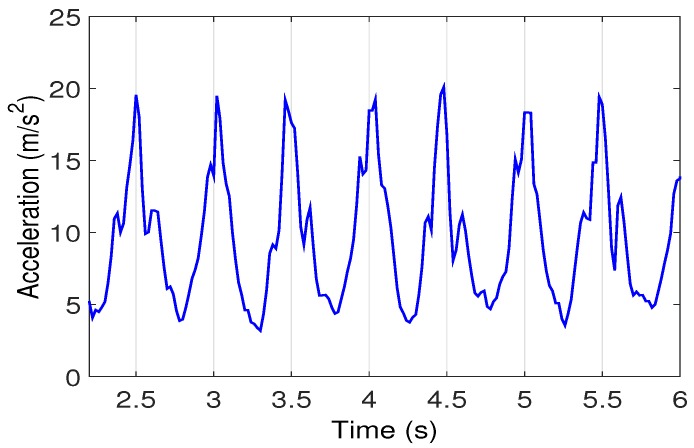
Recordings from accelerometers while pedestrians are walking.

When the pedestrian walks horizontally, periodical variations can be detected from accelerometer readings, as shown in Figure 2. It appears to be an approximate sinusoidal curve. By performing peak detection with a given threshold, pedestrian steps can be recognized in real time [10].

#### 3.2.2. Step Length Estimation

There are two ways to estimate step length: one is to set a fixed step length during the walking process according to the characteristic of pedestrian’s body; the other is to establish a dynamic calculation formula of step length based on humans’ walking features. The former is easier to be implemented but has a larger error while the latter is more complicated with a higher accuracy. The widely used dynamic approaches are listed as follows: the Scarlet approach is adopted and *k* is set to 0.81 in this paper, according to the experimental results by Pratama and Hidayat [23].

(1) Weinberg approach, the authors found that hip displacement in the vertical direction was proportional to step length to some degree [12]. Step length can be calculated as Equation (2) illustrates:
  (2) $$L_{winberg}=k*\sqrt[{4}]{a_{max}-a_{min}},$$
  where $a_{max}$ and $a_{min}$ are the maximum and minimum value of the acceleration in vertical direction during a step, and *k* is a constant.
(2) Kim approach, proposed an experimental equation, which represented a relationship between step length and average acceleration that occurred during a step [10]. The step length is calculated as Equation (3):
  (3) $$L_{kim}=k*\sqrt[{3}]{\frac{\sum_{i=1}^{N}\left|a_{i}\right|}{N}},$$
  where *N* represents the number of acceleration sampling points during a step, $a_{i}$ is the acceleration in one sampling process, and *k* is a constant.
(3) Scarlet approach, which improved the Weinberg approach, solved the variation problem deriving from different pedestrians or different paces and stride lengths of a same pedestrian [24]. The step length is calculated as Equation (4):
  (4) $$L_{Scarlet}=k*\frac{\frac{\sum_{i=1}^{N}\left|a_{i}\right|}{N}-a_{min}}{a_{max}-a_{min}},$$
  where *N*, $a_{i}$ represent the same as in Equation (3), $a_{max}$ and $a_{min}$ have the same meaning of these in Equation (2), and *k* is a constant.

#### 3.2.3. Walking Direction Estimation

Gyroscopes are the most frequently used inertial sensors in identifying walking direction because they can precisely measure the angular velocity of a moving object and are independent of interference from surroundings. By integrating gyroscope data, the turning angle Φ during a step can be obtained as Equation (5):

(5) $$\Phi =\sum_{i=1}^{N}\omega_{i}t,$$

where *N* represents the number of sampling points during a step, $\omega_{i}$ is the angle velocity in one sampling, and *t* is the sampling interval.

By adding the turning angle at each step to the previous walking direction, a new direction is determined. However, due to an inherent drift error of gyroscope, the accuracy of walking direction will decay if this error is not eliminated.

### 3.3. Landmarks Store

In this paper, we make the following assumptions about the indoor environment:

- APs are placed inside the rooms or above the corridors, and the WiFi signal can cover the pedestrian area.
- Angles of indoor corners are all right angles; between every two adjacent corners is a straight path.

On the basis of the above assumptions, certain locations, such as building entrances, are picked as the origin to build a coordinate frame. Some other special locations with definite position information are defined as ’landmarks’ that can be used to calibrate PDR positioning. Detailed definitions and identification procedures of these landmarks are described as follows.

#### 3.3.1. AP within Visual Range

APs have been widely deployed in indoor environments. Theoretically, the distance between pedestrians and AP can be estimated by RSS measurement on the premise that accurate wireless signal transmission model is established. Indoor environment is very complex, and the existence of reflection, refraction, scattering and a variety of segmentation loss make it difficult to realize accurate RSS measurement. However, when a pedestrian is approaching an AP within a visual range, the variation of RSS readings becomes relatively stable.

**Figure 3:**
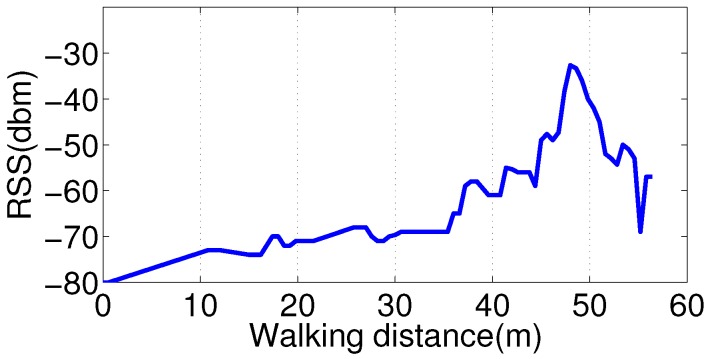
RSS variations when passing by an AP.

Supposing that a pedestrian is walking along a path under the visual range of an AP, RSS readings will present an obvious trend with a peak. Obtained from a smartphone when we walk past an AP above the corridor, all RSS readings are drawn in the form of a curve in Figure 3, and the coordinate of the AP is (48, 0). According to this phenomenon, we can take the projection point of the AP on a pedestrian’s walking path as a landmark.

#### 3.3.2. Door with an AP Inside

**Figure 4:**
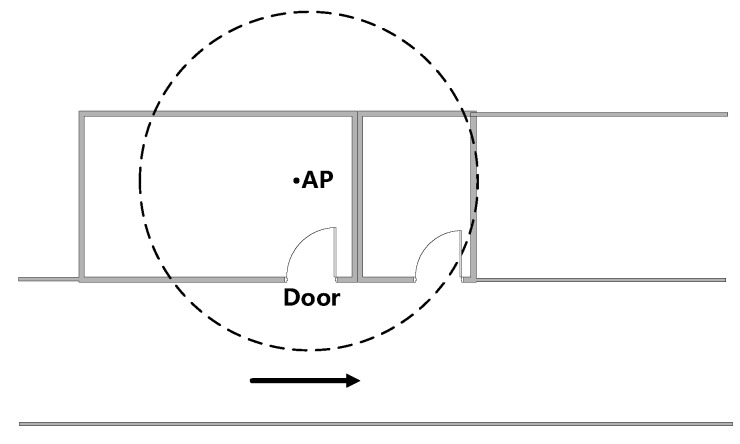
Door with an AP inside.

**Figure 5:**
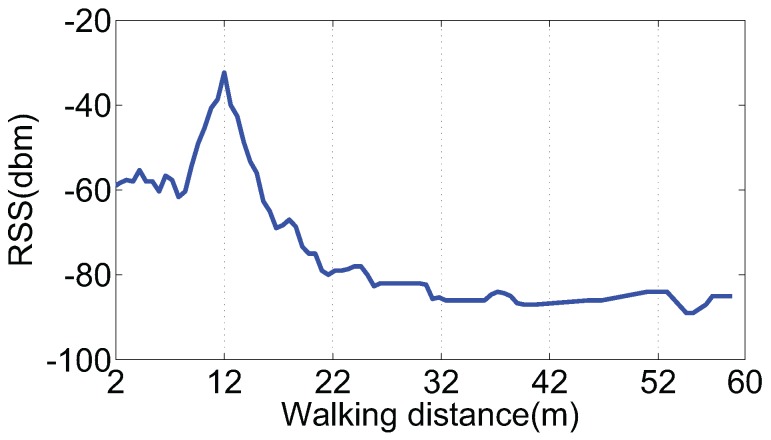
RSS variations when passing by a door.

As Figure 4 shows, when a pedestrian walks past a door with an AP inside, measured RSS also presents an obvious trend with a peak because the shadowing effects caused by doors and walls are different. We carried out an experiment to collect RSS readings when passing by a door. The RSS curve is shown in Figure 5, and the coordinate of the door is (12, 0). Thus, a door with an AP inside can also be regarded as a kind of landmark.

#### 3.3.3. Indoor Corners

Indoor corners contain explicit position and direction information, and can be regarded as another kind of landmark. To identify the corner on a floor plan, the first step is to recognize the turning actions of pedestrians. Considering that gyroscopes can only provide relative directions, we use a slide window and a threshold to determine whether turning happens.

**Figure 6:**
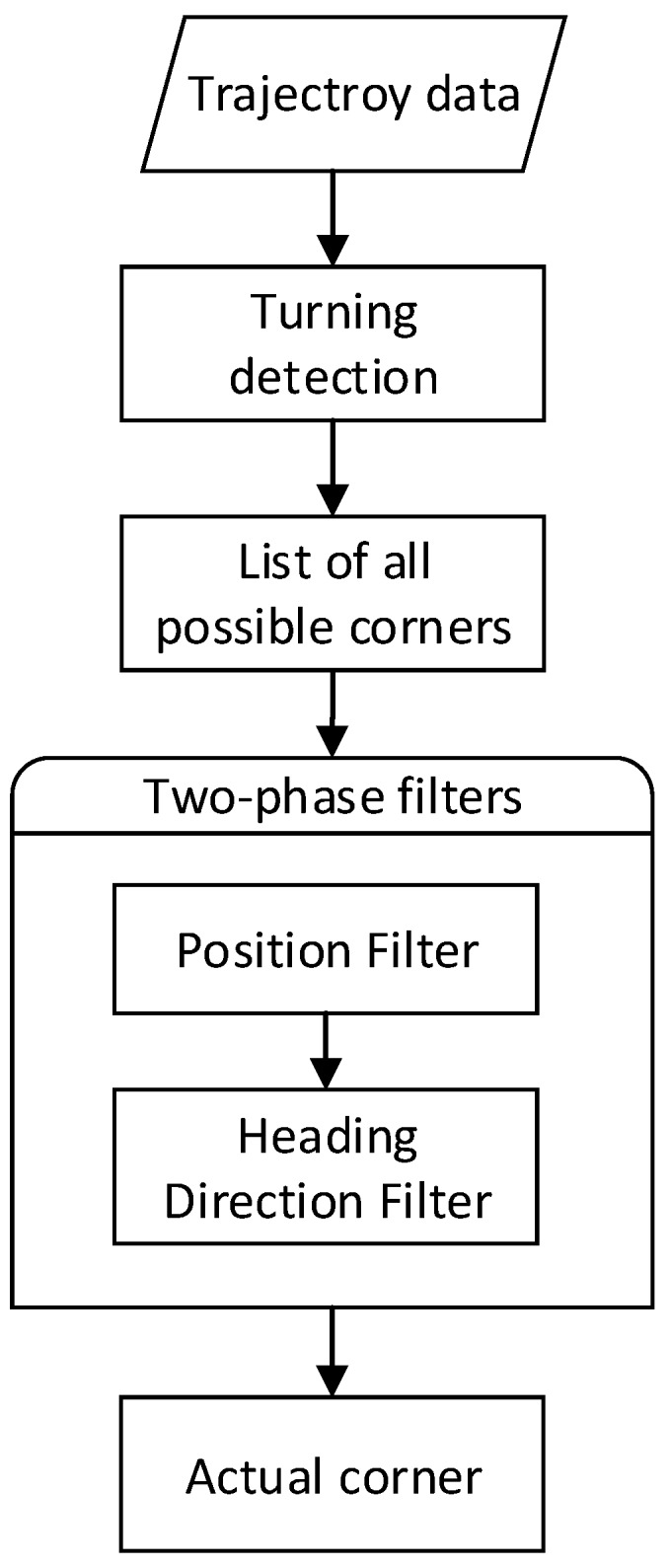
Flow chart of a corner-matching algorithm.

When a turning action is recognized, the following step is corner matching. In this step, we compare the current position and heading direction estimated by PDR to all corner positions and directions in the landmark database. If the deviation is under a certain threshold, we consider it a successful matching. The flow chart of the corner-matching algorithm is shown in Figure 6.

#### 3.3.4. Walls

**Figure 7:**
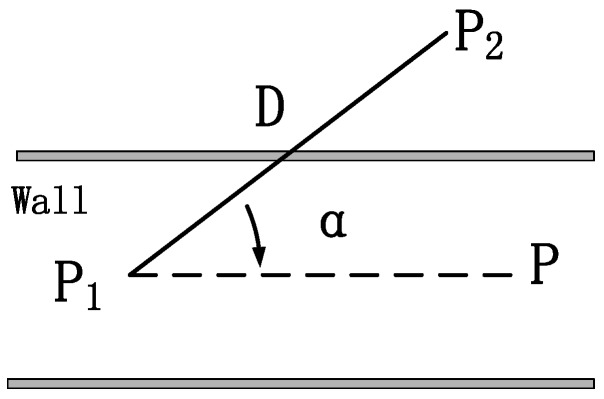
Trajectory intersects with a wall.

Walking across a wall can never happen while a pedestrian is walking in the real world. If the walking trajectory intersects with a wall, as Figure 7 shows, it means that the pedestrian’s position is wrong and needs to be calibrated. Therefore, a wall can be regarded as a special kind of landmark. For an indoor wall, we see it as a line segment for storage, when the pedestrian path and a line segment intersects, we consider that the trajectory drifts.

#### 3.3.5. Study on RSS Peak Detection

For RSS peak detection, we first use a moving average filter to eliminate the indoor noise. The original RSS readings are smoothed with Equation (6):

(6) $$y_{k}^{\prime }=\frac{1}{m}\sum_{i=0}^{m-1}y_{k+i}\;k=1,2,3...,$$

where *y* is the original reading, $y^{\prime }$ is the result after filtering, and *m* is the filtering step.

When a pedestrian either passes through an AP-style landmark (as described in Section 3.3.1), or a door-style landmark (as in Section 3.3.2), peak detection is an indispensable work to sort out the key point as the location of such a landmark. In practice, we set a sliding window to help locate the peak point.

Because the proposed fusion scheme in this paper is supposed to be light-weight and suitable for real-time implementation on smartphone, we adopt a simple but efficient algorithm to detect an RSS peak, which is called a sliding window algorithm. To identify the peak point, we use a $RSS_{max}$ variable to maintain the present maximum value of RSS and keep updating it when a higher value is detected in the next step. Only if the $RSS_{max}$ variable remains unchanged after certain steps, the number of which is pre-defined as the sliding window, is the value kept in $RSS_{max}$ deemed as the real maximum RSS value. Meanwhile, the step, where the maximum of $RSS_{max}$ is obtained, is thought to be the RSS peak point. The detailed algorithm description is listed as Algorithm 1 shows.
**Algorithm 1:** RSS peak detection with slide window
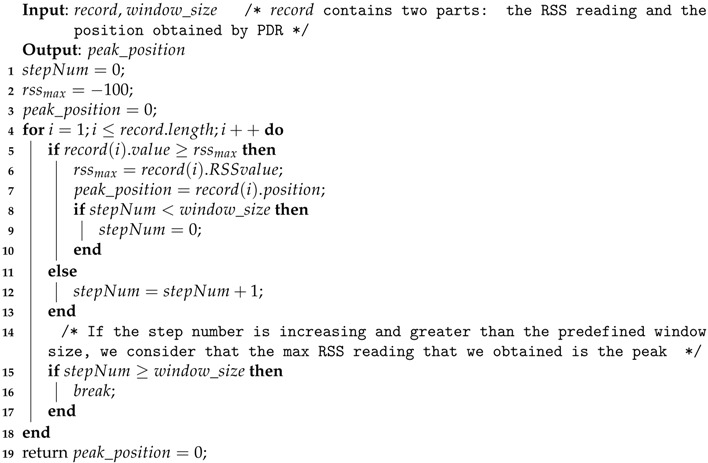


### 3.4. Landmark-Aided PDR (LaP) Positioning

After constructing a landmark database, we can use landmarks to revise drift in PDR for a better indoor positioning. A conventional calibration process is executed when a pedestrian passes by a landmark. According to study on RSS peak detection, our proposed algorithm cannot recognize this landmark until a pedestrian’s walking steps equal the sliding window. As soon as the peak is determined, the position calibration of PDR can be made.

#### Position Calibration

Position calibration procedure varies with landmarks, and the details are as follows:

(1) When a pedestrian walks past an AP or door kind of landmark *L*, the position of *L* is $P_{L}$, the position derived from PDR is $P^{\prime }$, and the position to update is *P*. If a match succeeds, then $P=P_{L}$.
(2) When a pedestrian walks past a corner kind of landmark *L*, the position of *L* is $P_{L}$, the position derived from PDR is $P^{\prime }$, the position to update is *P*, and then $P=P_{L}$. In addition, *L* contains direction information $\Phi^{\prime }$, the direction to update is Φ, and then $\Phi =\Phi^{\prime }$.
(3) When the trajectory intersects with a wall, as Figure 7 shows, the position from PDR before and after the intersection is $P_{1}$, $P_{2}$, the included angle between the trajectory and the wall is *α*, the step length is *D*, the position to update is *D*, and then we get the equation as follows:
  (7) $$P=P_{1}+D\left(\begin{array}{c} \sin\alpha \\ \cos\alpha \end{array}\right).$$

## 4. Evaluation

In this section, real experiments are conducted to evaluate the performance of LaP. Experiment results are illustrated and analyzed, and a comparison is made between PDR and LaP. We also make a comparison among LaP and two kinds of WiFi, and PDR and floor plan fusion: in the following, we will use the abbreviations PF-based [21] and EKF-based [22] to indicate the two approaches, respectively. The reason why the WPL-based approach [20] is not considered in this section is that we find it difficult to obtain an accurate pass-loss model of this indoor environment.

### 4.1. Experiment Set-Up

We develop an Android app to collect location data during the experiments and then analyze the data with MATLAB (R2016a, The MathWorks, Natick, MA, USA). The device involved in the experiments are two smartphones running an Android 6.0 operating system (Google, San Francisco, CA, USA). The phone models are HUAWEI Honor 8 (4 GB Ram/64 GB Rom) (Shenzhen, China) and HTC A9w (3 GB Ram/32 GB Rom) (Taoyuan, China). In the following, we will use “honor” and “htc” to represent these two phones, respectively.

**Figure 8:**
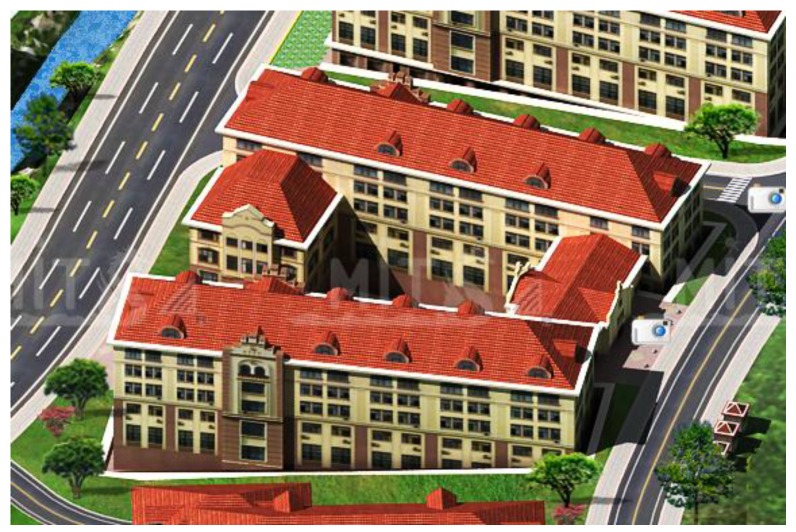
3D model of the experimental site.

**Figure 9:**
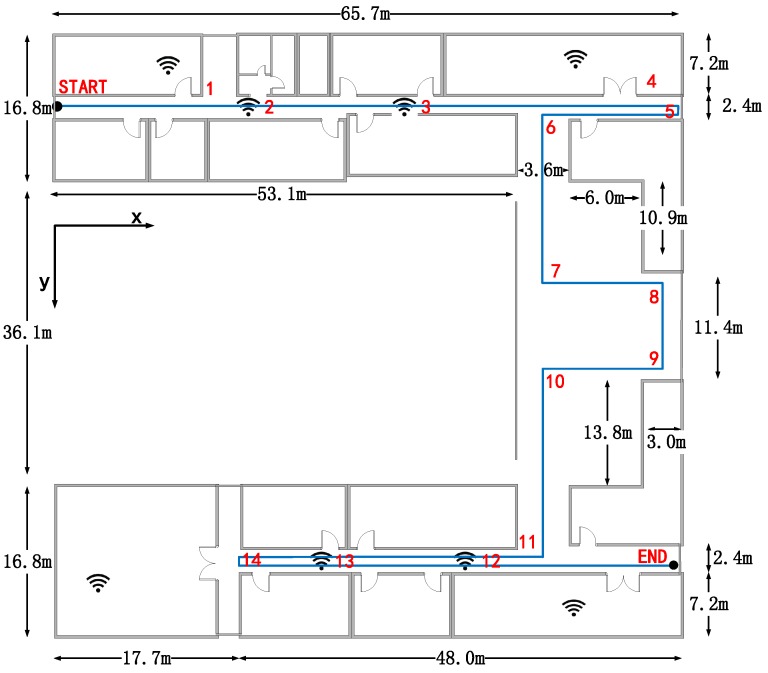
Floor plan of the building.

The experiments are conducted on the third floor in the College of Information Science and Engineering of our university. The flat size of the building is 65.7 m by 69.7 m, and Figure 8 shows the 3D model of the experimental site. Figure 9 shows the floor plan of the third floor, and the detailed building size information is marked in the figure. The blue broken line is the actual path that the pedestrians walk along during the experiment and the start point’s coordinate is set as the origin. We pick 14 landmarks that are marked in red in the figure. The coordinates and types of the landmarks are shown in Table 1.

**Figure 10:**
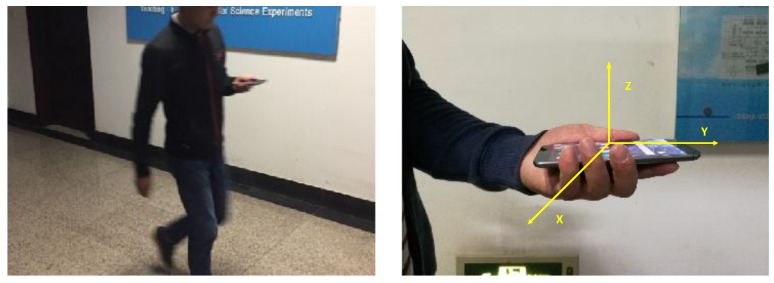
Attitude of the smartphone and the pedestrian’s gesture in the experiment.

There are three pedestrians to do the experiment, and we use letters J, Z, W to represent them, respectively. During the experiments, a pedestrian is supposed to walk along the pre-decided trajectory with the smartphone in hand. The attitude of the smartphone and the pedestrian’s gesture are shown in Figure 10. We select 16 reference points to record the pedestrian’s real position during the walks.

For the comparison of LaP and other WiFi, PDR, and floor plan fusions, we use the same smartphones and let all pedestrians do the experiments as well.

### 4.2. Experimental Results

#### 4.2.1. Comparison between PDR and LaP

**Figure 11:**
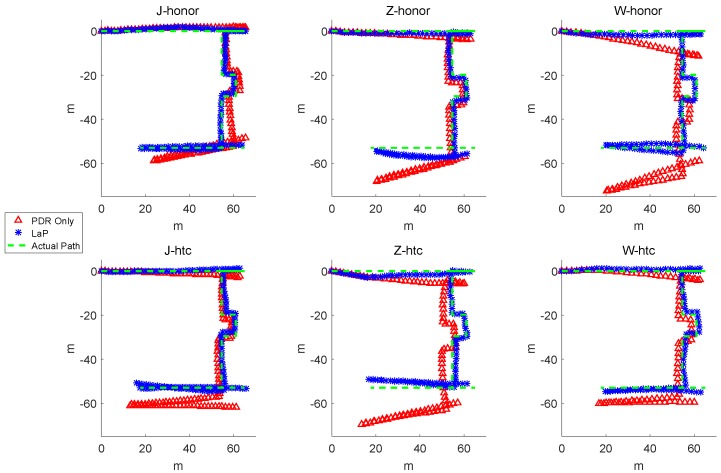
The actual path, the trajectories of PDR and LaP.

Figure 11 shows the actual path, comparing with the trajectories of PDR only and LaP from six experiments.

From the results, especially on the path between landmark 11 and the end point, the trajectories with the same smartphone of the three pedestrians show a similar bias. For pedestrian J, the trajectories of PDR are much closer to the actual path compared with the other two pedestrians. When using the HUAWEI Honor 8 smartphone model, pedestrian W generated a PDR trajectory with a large bias as well as pedestrian Z when using model HTC A9w.

On the micro level, when taking one trajectory into consideration (e.g., W-honor), we can see that, as the experiment goes on, PDR produces a large accumulated error for both distance and direction. In the first straight path, the trajectory of PDR only is obviously longer than the actual path. Moreover, there is a great angle drift in the clock-wise direction of PDR only. On the contrary, the trajectory of LaP is closer to the actual path.

We then focus on the positioning accuracy between PDR and LaP. As shown in Table 2, compared with PDR, the proposed approach performs much better, and it could achieve mean position error of 2.17 m and standard deviation of 1.34 m. The PDR is 6.27 m and 4.15 m. LaP reduces the localization errors by 65.6% compared with the PDR only approach

**Figure 12:**
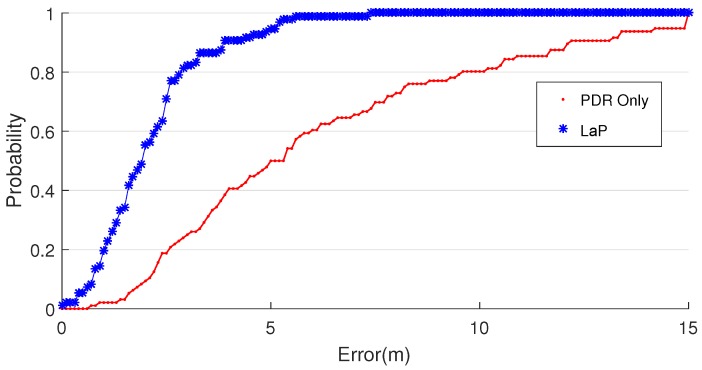
Localization error CDF.

**Figure 13:**
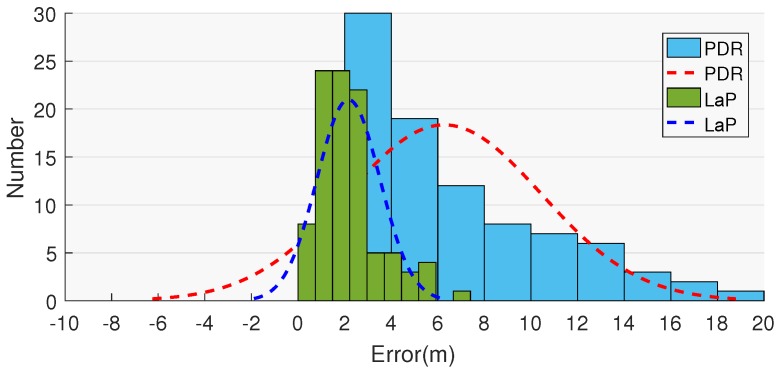
Location error distribution.

The location error CDF and distribution are demonstrated in Figure 12 and Figure 13. The CDF curve of LaP is far below the curve of PDR in the range of (0, 8).

#### 4.2.2. Comparison of LaP and Other Multi-Fusion Approaches

**Figure 14:**
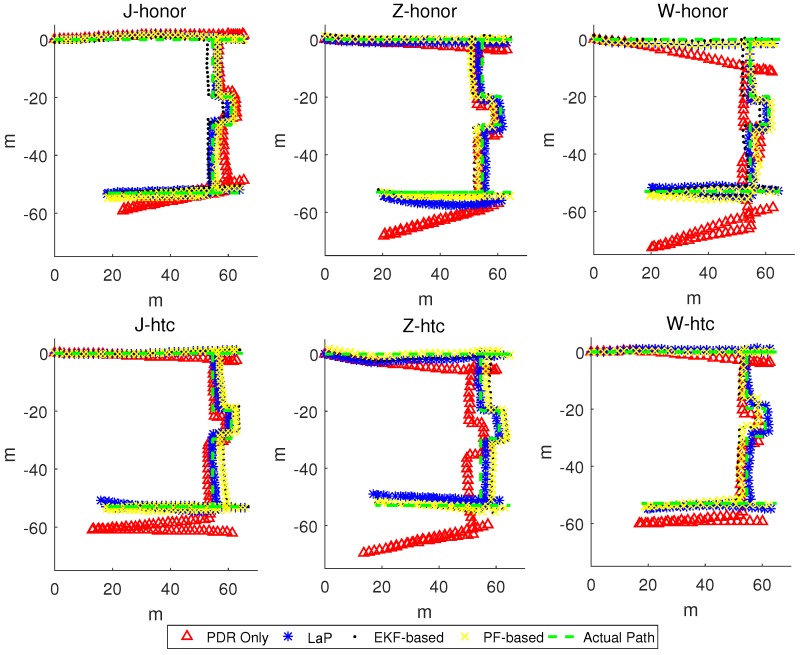
Trajectories of LaP and two other approaches.

Figure 14 shows the trajectories of PDR only, LaP, PF-based, and EKF-based approaches from six experiments. From the results, on the path between start point and landmark 6, and the path between landmark 11 and the end point, the trajectories of all three approaches are close to the actual path. There is a significant difference while walking from landmark 6 to landmark 11, and the proposed algorithm obtains a much closer trajectory to the actual path.

**Figure 15:**
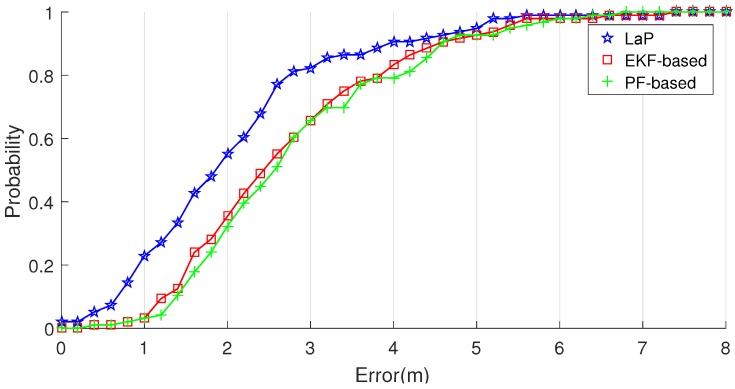
Localization error CDF of LaP and two other approaches.

**Figure 16:**
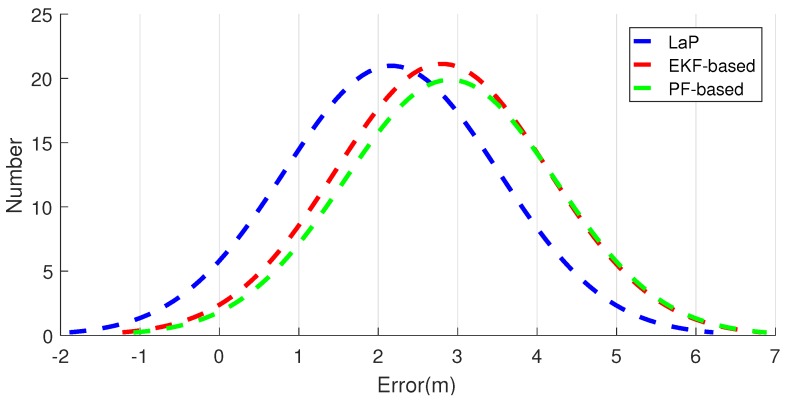
Localization error distribution of LaP and two other approaches.

We then focus on the positioning accuracy among LaP and other multi-fusion approaches. As shown in Table 3, compared with PF-based and EKF-based approaches, the proposed approach performs much better, while it could achieve mean position error of 2.17 m and standard deviation of 1.34 m. The location error CDF and distribution are demonstrated in Figure 15 and Figure 16. The distribution curve of LaP is much higher and narrower than that of PF-based and EKF-based approaches.

### 4.3. Discussion

Through analyzing the experiment results of PDR and LaP, we found analogous bias from the same smartphone, which means effects of one smartphone on different pedestrians are almost the same. In addition, compared to smartphones by themselves, pedestrians have a greater impact on the results. Even from one smartphone, for example, there is a a great difference among trajectories J-honor, Z-honor and W-honor. The proposed algorithm is designed to handle accumulated errors caused by both smartphones and pedestrians. Once a pedestrian passes by a certain kind of landmark, accumulated errors of the accelerometer and the gyroscope can be corrected well. Experimental results show that LaP effectively improves the localization accuracy of PDR.

**Figure 17:**
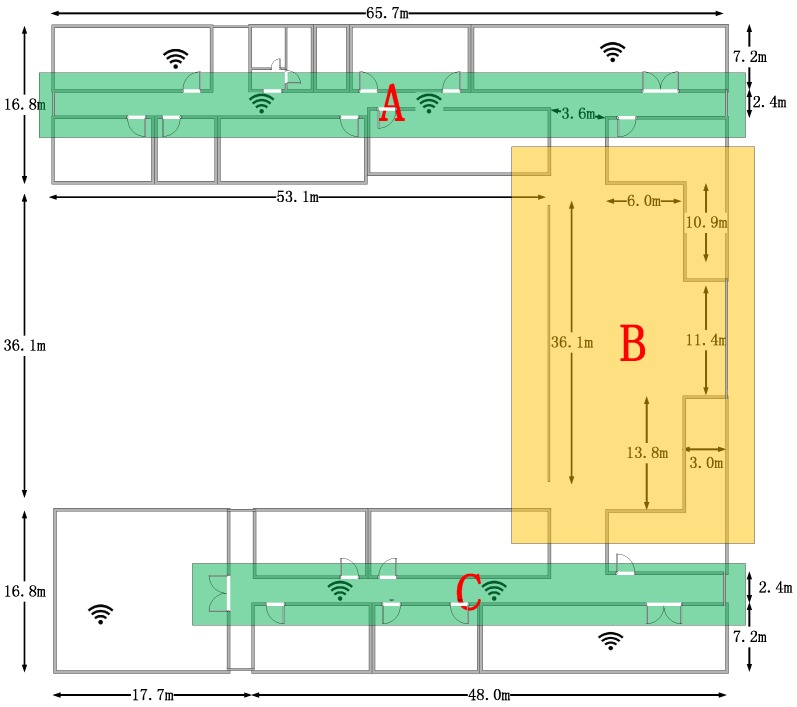
Path separation.

Figure 14 shows the trajectories of PDR only, LaP, PF-based, and EKF-based approaches from six experiments. We separate the whole path into parts A, B and C, as Figure 17 shows. From the results, we see that in parts A and C, all approaches work well. However, when pedestrians walk in the part B area, the location errors of EKF-based and PF-based approaches increase.

The reason why LaP can get a better result is that in part B, there is no access point coverage, and our approach takes full advantage of positions of indoor corners to correct the accumulated errors derived from the accelerometer and the gyroscope. The two-phase filters of the corner matching algorithm guarantee that we can get a relative accurate matching of the actual corner. These two multi-fusion approaches work badly in these areas because the fusion they adopt cannot be realized without WiFi information, and in the path in part B, the location accuracy largely depends on the performance of the original PDR algorithm. The experimental results show that, in AP-sparse indoor environments, LaP can still work well even if APs cannot cover all of the areas.

## 5. Conclusions

In this work, we have proposed a novel approach, LaP, for indoor positioning. LaP explores some special locations of indoor environments, which can be recognized by observing value variations of inertial sensors on smartphones. These special locations, called landmarks, are exploited to correct system cumulative errors. Finally, we develop an Android app for real experiments in a designated testbed to compare LaP with a PDR only approach and other multi-fusion approaches. Experiment results show that LaP works well in AP-sparse environments and can effectively improve localization accuracy. Furthermore, LaP is a light-weight approach without any adoption of a fusion filter, which can be easily implemented in real time on smartphones.

In the future, we will focus on identifying more kinds of landmarks in indoor environments, such as stairs, elevators and so on, to support a better correction of PDR errors. More kinds of landmarks will be used to improve experiments, and coverage influence of landmarks will also be taken into consideration. In addition, we also find the performance of PDR shows a distinct difference between different placements of smartphones during a pedestrian walk, motivating us to utilize this potential indicator for further improvement.

## Figures and Tables

**Table 1:** Position of landmarks selected in the experiment.

Number	*X*-Axis (m)	*Y*-Axis (m)	Type
1	9.6	0	Door
2	25.8	0	AP
3	45.6	0	AP
4	60.9	0	Door
5	65	0	Corner
6	54.6	0	Corner
7	54.6	−19.8	Corner
8	61	−19.8	Corner
9	61	−29.6	Corner
10	54.6	−29.6	Corner
11	54.6	−53	Corner
12	46.8	−53	AP
13	34.8	−53	AP
14	17.7	−53	Corner

**Table 2:** Position errors.

Person & Device	Method	Mean Error (m)	Standard Deviation (m)
J-honor	PDR	3.854	2.008
	LaP	1.842	1.311
J-htc	PDR	3.755	2.335
	LaP	1.936	1.456
Z-honor	PDR	5.796	3.400
	LaP	3.027	1.566
Z-htc	PDR	8.555	3.493
	LaP	2.228	1.294
W-honor	PDR	11.832	3.569
	LaP	2.022	0.985
W-htc	PDR	3.849	1.740
	LaP	1.937	0.958
Total	PDR	6.274	4.152
	LaP	2.166	1.343

**Table 3:** Position errors of LaP and other multi-fusion approaches.

Person & Device	Method	Mean Error (m)	Standard Deviation (m)
J-honor	PDR	3.854	2.008
	LaP	1.842	1.311
	EKF-based	2.471	1.386
	PF-based	2.744	1.225
J-htc	PDR	3.755	2.335
	LaP	1.936	1.456
	EKF-based	2.947	1.379
	PF-based	2.890	1.244
Z-honor	PDR	5.796	3.400
	LaP	3.027	1.566
	EKF-based	3.049	1.376
	PF-based	3.264	1.097
Z-htc	PDR	8.555	3.493
	LaP	2.228	1.294
	EKF-based	2.701	0.939
	PF-based	2.896	1.429
W-honor	PDR	11.832	3.569
	LaP	2.022	0.985
	EKF-based	2.541	0.942
	PF-based	2.658	1.063
W-htc	PDR	3.849	1.740
	LaP	1.937	0.958
	EKF-based	3.114	1.681
	PF-based	2.971	1.683
Total	PDR	6.274	4.152
	LaP	2.166	1.343
	EKF-based	2.804	1.334
	PF-based	2.904	1.321
